# Stimulating maximum staff performance in cross-functional and multiracial work environments: personal perspectives

**DOI:** 10.11604/pamj.2022.41.278.33267

**Published:** 2022-04-06

**Authors:** Obinna Ositadimma Oleribe, Guglielmo Maria Trovato

**Affiliations:** 1 Best Health Consult, Limited Liability Company (LLC), Klamath Falls, Oregon, United States of America,; 2 The School of Medicine, University of Catania, Catania, Italy,; 3 The European Medical Association (EMA), Brussels, Belgium

**Keywords:** Worker satisfaction, dysfunctional systems, worker performance, work environment

## Abstract

In a world where there is added stress on healthcare systems, owing to the COVID-19 pandemic, we acknowledge that healthcare systems around the globe often run in a dysfunctional way at the best of times. In this article, we identify some of the issues that surround workers´ dissatisfaction including lack of open, accountable, transparent, and honest management. We examine the theories behind a more accountable work environment and examine the potential for improved worker productivity as a result of physical, emotional, mental, intellectual, and spiritual wellbeing. We conclude that there is a need for organizational leaders and administrators to support organizational justice through the expanded use of work team processes, whereby staff is involved in assessing organizational functionality and recommending improvements. Supervisory consideration, job variety, and perceptions of training have positive effects on job satisfaction, staff performance, and organizational commitment.

## Commentary

Work is viewed as an important aspect of quality of life, and being unemployed is associated with a higher risk of common mental disorders [1]. To enjoy the beneficial effects of work, people seek to work. Obtaining the ideal job is difficult in today´s competitive environment, which truly manifests the concept of survival of the fittest. Globalization, use the of internet technology and globalization of remote working following the COVID-19 pandemic have led to many job losses, severe competition for the available jobs, and mass exodus of people from highly impacted nations to the rest of the world. In the United States, COVID-19 increased the unemployment rate with job losses up to three times as large for non-remote workers, decreased hours of work and labor force participation, and had no significant impacts on wages [2]. These negative impacts on the labor market were more several among men, younger workers, African Americans, Hispanics and less-educated workers, thereby increasing labor market inequalities [2].

Employee performance at work is dependent on a number of factors. These maybe organizational-related factors (leadership style, management support, training culture, environmental dynamism and organizational climate), job-related factors (work packages, job significance, environment, autonomy, communication) and employee-related factors (employee motivation, discipline, skill flexibility, skill level, proactivity, adaptability and commitment) [3,4]. Moreover, work ethic affects worker job satisfaction and performance, because strong work ethics contribute to encouraging and sustained better worker job performance, while absence or weak work ethics results in discouraging and diminishing workers job performance [5].

Generally, people seek employment because it is expected by the system or family that they work; to improve quality of life, buy what they want, pay bills and debts; to improve their skills and capacities in their areas of interest; to complete their training requirements through internship in medicine, pharmacy, and laboratory sciences, for example, where this is mandatory; to maintain healthy life-style, fund vacations and recreational activities, and live in a healthy environment; to effectively occupy time and stay actively engaged, and to contribute their quota to global development and advancement, and make a tangible difference. Whatever maybe the reason for staff employment, staff apply for and or accept job offers because: it is the job of their dreams and they have waited for it for a while; the pay (wages and benefits) is good and maybe better than the rest; the position and or pay is an evidence of promotion or lifting in career; the work package is in line with what was studied or learned in school or vocational centers; the offer is prestigious and self-enhancing; it provides a soft landing pad for a novice in the system or a recent immigrant to a new nation; it is a stepping stone to the ideal or dream job, and keeps one busy until the desired is secured; the work environment or climate is good and friendly; and or it is the only job available or offered at the moment.

Whatever is the reason for working, looking for or accepting an offer, one can see that very few are actually initially highly motivated at the offer of an employment. Moreover, disrespectful treatments, little accomplishments, project confusion, lack of recognition, and communication breakdowns are known to have adverse effects on morale of the organizational work force. In addition, an unfavourable psychosocial working environment may pose a threat to the mental health of workers as several factors, such as increased work pace, more high-skilled jobs, and the increased use of information and communication technology have resulted in increasingly higher demands on the mental function of workers [1]. While there have been a variety of studies concerning worker motivation and productivity, any improvement in workers productivity could have significant financial and service impact for the organization and society at large [2-4]. Therefore, to get the best out of any employee, employers must stimulate, motivate, and sustain their engagement to work. They must also get them committed to organizational long-term vision and short-term goals, and support them to contribute maximally to judiciously earn their weekly, bi weekly, or monthly pay. To achieve this, employers and leaders or an organization must build a transparent environment. The various transparent management strategies, when put in place by the employers, ensure maximum productivity from their employees 24/7, all year round; and we suggest the following:

**Transparent and honest systems:** workers want to see their employers, directors, and supervisors open, accountable, transparent and very honest; and to operate in such a way that it is easy for others to see what actions are performed. Workers desire to see transparency and honesty in the implementation of organizational policies, guidelines, standard operating procedures, and all other rules of operation. Where this is not the case, workers conflicts, mushroom communication, cliques, and unhealthy associations may result mitigating productivity and general staff performance.

**Equity and fairness:** workers in all work environments, especially multi-racial cross-functional systems, expect a level playing field for all staff irrespective of race, gender, qualification and disability index. Any form of inequity and unfairness mitigates staff performance/productive, and engagement. Organizations are quick to insert in their vacancy announcement such disclaimers as, “this organization is an equal opportunity employer. All qualified applicants will receive consideration for employment without regard to status as a protected veteran or a qualified individual with a disability, or other protected status, such as race, religion, color, national origin, sex, sexual orientation, gender identity or age”. This is because, “it is illegal for an employer, employment agency or union to take into account a person's race, color, religion, sex (including gender identity, sexual orientation, and pregnancy), national origin, age (40 or older), disability or genetic information when making decisions about job referrals” [6]. Furthermore, “it is also illegal for an employer to recruit new employees in a way that discriminates against them because of their race, color, religion, sex (including gender identity, sexual orientation, and pregnancy), national origin, age (40 years or older), disability or genetic information” [6]. However, beyond these provisions and professed non-discriminatory organizational system, the applicant desire is to see this in operation at shortlisting, interviewing, selection, and onboarding into the organizational system. Employed and onboarded workers want to know and see that every staff member (irrespective of defined protected characteristics) operates in the same field with equity and fairness. Also, workers desire organizations where there are minimal favoritism and preference for the few at the expense of the rest. Workers want to see a fair, just and equitable promotion practices, and reward system that recognizes every good work.

**Healthy work environment:** a recent study from a medium-sized police department suggests that the work environment is a principal source of job satisfaction, regardless of the measure of the dependent variable employed [7]. Workers desire a healthy work environment where their health (physical, emotional, mental, intellectual and spiritual) is preserved, improved upon and actually made to flourish. Good ergonomics plus the provision of a gymnasium for physical exercises, and time out to refresh, recreate, and rejuvenate, are critical to physical health. Staff welfare, recognition, commendation, and positive feedback are vital to emotional and mental health of workers. In service trainings (onsite and offsite), mentoring, administrative/clinical/supportive supervision are key to intellectual health, and provision of opportunity or space for spiritual exercise like praying time and room is vital for spiritual health. Studies have shown that healthy work environment as demonstrated in a healthy community, workload, mental health, and job satisfaction played significant role in staff engagement and productivity within an organization [8]. Furthermore, higher job satisfaction and lower intentions to leave were found for individuals whose work environments complemented the creative requirements of their jobs. The requirement for a healthy work environment has spurred some organizations to make the work environment homely and a complete community providing most of the needs of the staff including refreshments. Investment made towards this is very profitable and will improve both staff performance and outputs thereby positively impacting on the bottom-line of the organization.

**Interesting work packages:** although according to Pareto principles, 20% of the staff deliver 80% of the profits of the organization, employee engagement in an interesting work package stimulates improved performance and staff retention. Workers who stay long in an organization to make a difference are usually involved in work packages that provide mental and intellectual stimulation. Also, workers like to see that they are learning new skills and expanding their capacities daily as they do their work. Although there may be other confounding or associated factors motivating staff such as salary and wages, workers know that these cannot provide sustainable motivation, but will always look out for those work packages that keep them awake because of the levels of challenges they generate. Therefore, to keep staff motivated and engaged, identify their core areas of interest and assign them to tasks that will stimulate them, engage their five plus one senses, and keep them mentally aroused and occupied.

**Significant work packages:** one of the factors to ensure a work package is interesting is its contribution to the society [6]. Majority of workers desire to work on solutions and processes that will make a difference in the society. Therefore, work packages that contribute to a better community and society are motivational to workers and facilitate better performance. These significant work packages help the workers have real impacts and evidence of their work in years to come. Leadership style exhibited by organizational managers can also make any and every work package significant as they focus on the impact of all work packages including the mundane ones to the value chain. For instance, although developing a vaccine against COVID-19 is really important, the scientists who achieved this may not successfully deliver if the office/laboratories were not properly cleaned and disinfected, if the power supply were not paid for and on time, if the internet facility failed at critical moments, and or if the equipment were not serviced and maintained to deliver to the highest level of accuracy.

Everybody in the value chain can be made to see the impact and significance of their contributions to the success of the vaccine trials and development. In a study among state workers in the United States of America, workers appreciate having freedom and autonomy, like their jobs and the sense of achievement, and welcome teamwork, but feel limited by poor supervision and management, poor communications, and insufficient budgets and staffing. It is critical to ensure that every one is given a task from which upon completion, they will have a positive sense of accomplishment, or made to see their work and assignment in a way that they enjoy this same sense of accomplishment at the completion of their work.

**Empowering wages and benefits:** Herzenberg two factor theory sees achievement, recognition, the work itself, responsibility, advancement, and growth as motivational factors; and company policies, supervision, relationships, work conditions, salary, status, and security as hygiene factors [9]. Although wages and benefits were seen as hygiene factors, these may actually be motivational for individuals who are yet to meet their psychological or basic life needs like food, shelter and clothing. It is motivational to all staff at different phases of their work lives and only becomes a hygiene factor when basic needs have been adequately met. Paying workers living wages, as and when due, allows for proper planning, ability to meet basic needs, and pay bills, and ensures staff engagement and sustained/long term employment with the system.

**Supportive and honest supervision:** supervision is critical to the performance of workers in any work environment as workers appreciate supervisors who help them achieve their personal and organizational goals. Studies have shown that leadership style, effective and supportive supervision, direct interactions with supervisors were identified as key to workers motivation and increased productivity. Therefore, eliminating unhealthy bureaucracy, ensuring improved and better supervision, and honest communication between supervisors and their staff will improve staff motivation and engagement resulting in enhance productivity.

**Organizational justice:** organizational justice is key to workers performance in a multiracial and cross functional system, as workers expect fairness, equity and justice in their work places. Organizational justice consists of distributive justice (the focus on the outcome of a decision) and procedural justice (the decision-making process that leads to the outcome). Organizational justice can also be looked as distributive justice, procedural justice and interactional justice [10]. Inadequate organizational justice resulting from unfair practices, nepotism or inequity have been found to be a leading predictor of job stress and variants of organizational commitment. Distributive, procedural, and interactional justice have a significant impact on self-efficacy, satisfaction and performance [10]. Moderate to high sense of equity bring about improved work environment and better staff engagement. Ensuring organizational justice is therefore commensurate with a better, healthier work environment with improved staff performance. The issues related to workplace bullying, in any social context, need a careful appraisal and appropriate and timely solutions.

**Conclusion and recommendations:** there is the need for organizational leaders and administrators to support organizational justice through expanded use of work team processes, whereby staff are involved in assessing organizational functionality and recommending improvements. Studies revealed that supervisory consideration, job variety, and perceptions of training had positive effects on job satisfaction, staff performance, and with organizational commitment. As high job demands, low job control, low co-worker support, low supervisor support, low procedural justice, low relational justice and a high effort-reward imbalance result in stress related diseases in work place, putting in place measures to minimize these will be to the benefit of the workers and organization as healthier stress-free staff will result in increased productivity.
